# The Effects of Exercise on Appetite in Older Adults: A Systematic Review and Meta-Analysis

**DOI:** 10.3389/fnut.2021.734267

**Published:** 2021-11-18

**Authors:** Sarah Hubner, Julie Blaskewicz Boron, Karsten Koehler

**Affiliations:** ^1^Department of Gerontology, University of Nebraska Omaha, Omaha, NE, United States; ^2^Department of Sport and Health Science, Technical University of Munich, Munich, Germany

**Keywords:** exercise, appetite, aging, leptin, satiety

## Abstract

**Background:** The effect of physical activity and exercise on hunger and satiety has been well-studied in younger adults, but the influence of aging is less understood. While some evidence suggests that acute bouts of exercise induce a compensatory eating drive, long-term activity may improve satiety sensitivity. The objective of this study was to investigate the effects of exercise on appetite in older adults.

**Methods:** We systematically reviewed available literature investigating the effect of exercise on appetite in older adults adults (CRD42020208953). PubMed, PsycINFO, Academic Search Complete, the Sports Medicine & Education Index, and Web of Science, were searched for peer-reviewed articles published in English with no date restriction. Included studies implemented a primary exercise or physical activity intervention with a control group, on a generally healthy population ≥60 years of age. Selected studies included at least one appetite outcome. Risk of bias was assessed using the 11-point Physiotherapy Evidence Database (PEDro) tool. Standardized mean difference summary statistics (Hedge's g effect sizes) and 95% confidence intervals were reported.

**Results:** We identified 15 reports (13 studies) which met all inclusion criteria (5 resistance training, 3 aerobic, 6 mixed modalities). Studies included 443 participants (Age = 68.9 ± 5.2, 82.3% female) and had generally “good” bias scores (PEDro = 6.4 ± 0.88). Random effects meta-analyses revealed that the exercising group showed statistically significant reductions in glucose [SMD = −0.34 (95% CI: −0.67, −0.02), *p* < 0.05, PEDro =6.4 ± 0.45] and leptin [SMD = −0.92 (95% CI: −1.28, −0.57), *p* < 0.00001, PEDro = 6.2 ± 0.75].

**Discussion:** This systematic review revealed that exercise and physical activity may modulate resting hunger and satiety in older adults. Decreases in fasting leptin and glucose hormones suggest that exercise promotes satiety sensitivity in adults aged 60+. This review highlights that engaging in exercise and activity programs may provide a meaningful avenue for improving chronic and functional disease burden in later life by promoting appetite control and balanced energy intake. Recommendations for future research include investigations of appetite in response to varied exercise modalities within more diverse and representative samples of older adults.

## Introduction

The number of older adults worldwide is projected to more than double by 2050, with those aged 65 and older reaching 1.5 billion within the next three decades (1). This demographic shift is happening concurrently with changes in work and lifestyle, specifically a global trend toward sedentary behavior (2). Overall, this inactivity negatively affects health, directly relating to a higher prevalence of chronic cardiovascular and metabolic disease (2). As such, physical activity and exercise are promoted to ameliorate physical, psychosocial, emotional, and cognitive wellness (3, 4). Worldwide physical activity guidelines recommend that older adults engage in moderate to vigorous-intensity aerobic activity a minimum of three times weekly, irrelevant of chronic disease status; guidelines also suggest integrating strengthening activities and multicomponent balance and fall prevention exercises and limiting sedentary time (4). Still, despite these recommendations, up to 70% of adults in developed countries report insufficient physical activity (2, 5). Low levels of physical activity and exercise ultimately increase disease risk, morbidity, and all-cause mortality (2).

Comorbidity is often compounded by overweight, obesity, and frailty, which are complex issues in older adult populations given age-related changes in body composition, adiposity, and appetite (2, 6–8). Exacerbated by low activity, older adults may experience unique weight-related challenges due to physiologically-driven, natural redistribution and increases in subcutaneous fat (6, 7). Increased adiposity can be concomitant with sarcopenia, the loss of muscle mass and strength associated with aging (6). Consequentially, efforts to *lower* body mass can affect poor nutrition and simultaneous fat and muscle loss, diminishing the prospective benefit of weight reduction (6, 7, 9). Further, age-related declines in metabolic and sensory systems, particularly those related to olfaction and gustation, can cause an “anorexia of aging” characterized by reduced eating drive and energy intake (10–12). Efforts to *maintain* body mass can also be attenuated by both undernutrition and malnutrition, precipitating sarcopenia and frailty (8, 12). Ultimately, biological changes complicate weight control in older adults, and management of energy intake remains key to mitigating morbidity and mortality (6, 7, 9, 12).

Across populations, physical activity and exercise are fundamentally associated with health outcomes and behaviors, extending to the management of body composition and energy intake (13). In young adults, it has been documented that physical activity and exercise may induce a compensatory response in energy intake, which can impact the long-term success of fitness regimens (13–16). Evidence from this population has demonstrated that, while acute exercise causes a brief, transient decrease in appetite, it likewise stimulates the release of orexigenic hormones and reduces anorexigenic processes, ultimately upregulating hunger and eating drive (13–16). Notwithstanding these acute hormonal changes and the associated physiological response, some research has failed to demonstrate the expected increase in post-exercise energy intake (13, 17). However, the effects of exercise are dependent on many individual factors, therefore this absence may at least partially stem from the mix of populations and methods included across research (18, 19). This may also be a consequence of relying on self-report methods, primarily diet logs, for assessment (17, 20, 21).

Considering chronic exercise and physical activity, evidence from long-term studies in young adults indicates that improvements in body composition and function result in enhanced satiety sensitivity, thus promoting metabolic balance and relative appetite control (18, 19, 22, 23). Further, sustained increases in activity have been evidenced to alter fasting levels of appetite-regulating hormones and metabolites (e.g., leptin, glucose, insulin, ghrelin, adiponectin) and heighten post-prandial satiety (13, 24). This suggests that fidelity to regular activity programs improves appetite control, helping to regulate accompanying increases in hunger drive (13). Prolonged exercise has also been linked to increased energy and protein intake, as well as improved muscle maintenance in aging populations, both of which can help mitigate adiposity, sarcopenia, and downstream disease risk (25–28). However, like acute exercise, there are bodies of opposing literature suggesting no effect of long-term physical activity on appetite, or that these relationships are inconsistent (13, 21, 29–32). Similarly, this is likely attributable, in part, to population and methodological variations between studies.

Overall, the relationships between exercise, physical activity, and appetite are poorly researched in older adults, with a marked paucity of aging-specific data (13, 17, 30). Previous research (3, 9, 13, 17, 21, 22, 27–36) on the effects of exercise and physical activity on appetite in aging populations is inconsistent and appears to be unclear. The purpose of this systematic review and meta-analysis was to assess the existing literature on exercise and physical activity interventions in older adult populations, with the aim of better understanding how they may impact hunger and satiety in aging groups. The authors hypothesized that activity and exercise-induced consequences of hunger, satiety, and compensation differentially affect aging adults. Additionally, despite the potential for negative outcomes, exercise and physical activity might lead to net improvements in appetite-regulating pathways and resultant health. The goal of this research was to elucidate known effects and identify areas for future investigation in aging populations.

## Method

A systematic literature review and meta-analysis were performed following the guidelines set forth by the Preferred Reporting Items for Systematic Reviews and Meta-Analyses [PRISMA, Supplementary Tables 1, 2 (37)]. Scope, methods, and aims for the study were decided upon a priori by the study team and are presented in a systematic review protocol (Prospero, 2020 CRD42020208953, *Blinded for Review*).

### Literature Search Strategy

Electronic database searches were conducted in PubMed, APA PsycINFO, Academic Search Complete, the Sports Medicine & Education Index, and Web of Science. The search of Web of Science included the Science Citation Index Expanded, Social Sciences Citation Index, Arts & Humanities Citation Index, and Emerging Sources Citation Index. Database searches were conducted in July 2020 and June 2021. Search results included all available reports from inception of the respective database to 5 June 2021.

The search strategy combined the following relevant terms: (1) *Title*: older^*^, elder^*^, senior^*^, or geriat^*^, AND (2) *Title*: exercise[Mesh]/exercis^*^, exert^*^, energ^*^, fitness^*^, activit^*^, isometric, anaerobic, aerobic, or isotonic, AND (3) *Title, Abstract, or Topic*: leptin, ghrelin, peptide tyrosine tyrosine, peptide yy, pyy, appetite, hunger, adiponectin, satiet^*^, GLP-1, GLP 1, glucagon like peptide 1, glucagon-like peptide 1, CCK, cholecystokinin, NPY, neuropeptide Y, AgRP, agouti-related peptides, orexin, pro-opiomelanocortin, pro opiomelanocortin, CART, cocaine-and-amphetamine regulated transcript, cocaine and amphetamine regulated transcript, CRH, corticotropin-releasing hormone, corticotropin releasing hormone, diet^*^, eating, ate, consum^*^, or food^*^.

Search terms were also used to exclude studies with titular indications of younger participants or non-human subjects. Specific relevant terms included: (1) *Title*: adolesce^*^, young adult^*^, youth, child^*^, or infant^*^, OR (2) *Title*: mice, mouse, pig, or rat. Database limiters/filters were used to exclude non-English publications and gray literature where appropriate.

The title/abstract screen and the full-text screen were performed by two reviewers (SH and MG). Disagreement and ambiguities in eligibility criteria were resolved by a third reviewer (JB) and consensus among authors. Results from the database search were managed in EndNote X9 (38) and Zotero 5.0 (39) citation software. Deviating from the pre-specified protocol, hand searching was performed, consisting of assessment of (1) included articles' reference sections and (2) relevant reviews and meta-analyses, identified via the database search. Hand searching was completed to ensure review of potentially missing reports, including any additional manuscripts associated with the included articles. This was deemed necessary given the small number of reports, in addition to the identification of several pairs of articles based upon the same studies, discussed later in further detail.

### Study Selection Criteria

The specific research question for this systematic review and meta-analysis was “does exercise or physical activity influence hunger, appetite, or satiety outcomes in healthy adults aged 60 and older?” Studies on aging populations which implemented exercise or physical activity interventions and included hunger and satiety outcomes were included in this study. Considering the participants, interventions, comparators, and outcomes (PICOS) systematic review and meta-analysis design: (1) Participants were healthy adults 60 years and older. Studies which included participants with obesity and/or sarcopenia were included, and additional sub-analyses were proposed for these reports. (2) All studies included physical activity and/or exercise interventions. Interventions were classified as aerobic, resistance, or mixed modality. (3) At minimum, studies included an exercise-only group/condition and a control group/condition. To aid group comparison, controls were either a unique control group or the intervention group at rest, normal activity, or low activity (e.g., walking). (4) Included reports presented outcomes related to hunger-regulating hormones (e.g., ghrelin, leptin), compensatory eating (e.g., energy intake), and/or satiety (e.g., appetite). Although not explicitly indicated in the original PROSPERO registration, insulin/glucose was added as an outcome due to its frequency of reporting. Insulin and glucose are important signals within the orexigenic cascade, so inclusion is warranted (13, 24). In order to retain the pre-specified search strategy, additional database searching for these outcomes was not conducted; however, if studies identified by the original search included these measures, they were considered for inclusion. Studies were restricted to articles published in peer-reviewed journals in English. Given the limited subject data, most study designs were eligible for consideration with minor exceptions, detailed subsequently.

Studies were excluded if non-human subjects or populations younger than 60 years were included. Additionally, reports with unclear outcomes or those that were qualitative, gray literature (e.g., book chapters, dissertations, etc.), case studies, or unpublished/incomplete manuscripts were ineligible. Reports that included persons with appetite or activity-modulating health/physical conditions (e.g., diabetes, eating disorders, joint replacements) were excluded. Similarly, studies with interventions which included modifying lifestyle programs, supplements, or other similar components were excluded in absence of representative exercise-only and control-only groups. Interventions with maintenance/management diets based on participants' normal energy intake were eligible for inclusion as they were not considered to be ‘modifying' programs.

Although previous systematic reviews have been conducted examining concepts similar to this question, the scope and goals of these reviews differed significantly and, by result, their search criteria varied considerably compared to the present study (13, 35, 40). Further, this review specifically aims to provide a summary of available research on the relationships between exercise, physical activity, and appetite within a reasonably healthy older adult population.

### Data Extraction

Data was extracted by a single reviewer (SH). For studies that met all criteria, the following data were extracted: (1) sample (number of participants, mean age, proportion female); (2) intervention [exercise(s) and control(s)]; (3) study timing (length of study, number/frequency measures); and (4) hunger and satiety measures (pre- and post-test means, changes from baseline (change score), standard deviations, and correlation coefficients, when reported). In studies where more than one exercise intervention group was reported, summary statistics were combined and evaluated as a single group in alignment with Cochrane recommendations, where *N* is the number of participants, *M* represents mean, and *SD* is standard deviation (41):


$$\begin{array}{l} N_{Total}=N_{1}+N_{2}\\ M_{Total}=\;\frac{N_{1}M_{1}+N_{2}M_{2}}{N_{1}+N_{2}} \end{array}$$

$$\begin{array}{l} SD_{Total}=\sqrt{\frac{\left(N_{1}-1\right)SD_{1}^{2}+\left(N_{2}-1\right)SD_{2}^{2}+\frac{N_{1}N_{2}}{N_{1}+N_{2}}\left(M_{1}^{2}+M_{2}^{2}-2M_{1}M_{2}\right)}{N_{1}+N_{1}-1}} \end{array}$$

In reports where pre- and post-test results were reported, the following equations (41) were used to calculate, *M* and *SD* of the change score, where *r* represents the correlation coefficient between baseline and endpoint values:


$$\begin{array}{l} M_{Changes}=M_{Endpoint}-M_{Baseline}\\ SD_{Change}=\sqrt{{\left(SD_{Baseline}\right)}^{2}+{\left(SD_{Endpoint}\right)}^{2}-(2*r*SD_{Baseline}*SD_{Endpoint})} \end{array}$$

A convenience estimate of *r* = 0.5 was used in this study due to insufficiently reported data (14, 41). For studies presenting additional incomplete outcome data, procedures for estimation of missing data were performed (42), where necessary.

### Risk of Bias

Two reviewers (SH, MG) independently assessed each article for risk of bias without blinding to authorship or journal. Disagreements in scores across reviewers were resolved by consensus and inclusion of a third reviewer as necessary (JBB). The Physiotherapy Evidence Database (PEDro) risk of bias tool was utilized for study assessment (pedro.org.au).

### Data Analysis

Data management and summary statistics were conducted and produced in IBM SPSS Statistics 27.0 (43). Meta-analyses and associated data visualizations were conducted and produced in Review Manager (RevMan) (44). The statistical R package “metaphor” via MAVIS: Meta-analysis via Shiny was used to review meta-analyses, assess publication bias, and produce funnel plots, as appropriate (45). Mean effect summary statistics were represented by standardized mean differences (SMDs) and 95% confidence intervals (CIs). Treatment effects were measured as the net change in outcome values at baseline and endpoint. Individual and pooled Hedge's g effect sizes (SMDs) for small sample bias with random effects models were calculated. Random effects models were favored due to variance in the studies. SMD was used to judge the magnitude of the effect. Studies were meta-analyzed if there were three or more reports with valid data. The *I*^2^ test for heterogeneity was used with standard cutoffs of <25% indicating low heterogeneity, 25–50% representing moderate heterogeneity, and >50% indicating high heterogeneity. Sensitivity analyses were used in all meta-analytic calculations to assess robustness. Sensitivity analyses were conducted by removing one study at a time to assess for the effects of sample size, individual effect sizes, and study quality (risk of bias), where appropriate. Where possible, sub-analyses were used to examine studies which only included either healthy-weight or overweight/obese participants. For significant pooled effects, the funnel plot, rank correlation test for funnel plot asymmetry, and Rosenthal's fail-safe N were assessed to test for publication bias (42). All alpha levels were set to *p* < 0.05 a priori.

## Results

### Study Identification

**Figure 1:**
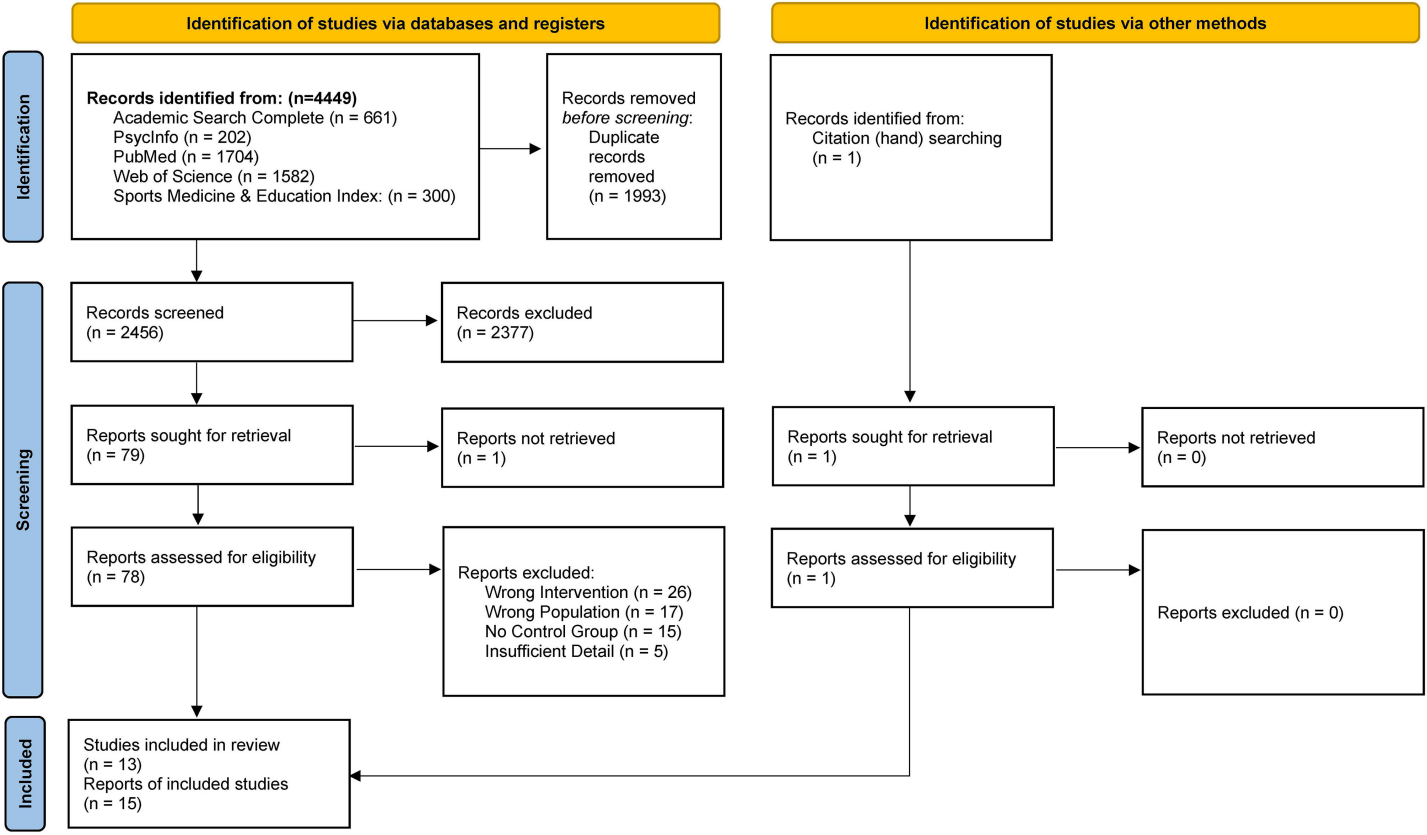
PRISMA Flow Diagram of study selection (37).

The literature search strategy accumulated 4,449 total titles. After duplicate deletion, 2,456 unique articles were identified for preliminary screening. Title and abstract evaluation identified 79 potential manuscripts for full text screening. After screening full articles, 19 reports met all inclusion criteria (46–64). One additional article was identified via hand searching (65). Corresponding authors of studies which included apparent duplicate or missing data (precluding full interpretation of the results), were contacted for clarification. No corresponding authors responded to these inquiries. Reports with duplicate data from included studies were retained, however, five studies which could not be interpreted without further detail were eliminated (59, 60, 62–64). Ultimately, 13 unique studies were represented by 15 reports and were included in analyses (46–59, 61). Four reports with duplicate data were consolidated and analyzed as two individual studies. Further, (1) Butterworth et al. and Nieman et al. (46, 55), and (2) Markofski et al. and Timmerman et al. (54, 61) were jointly analyzed. Timmerman et al. was included in this review as a relevant study duplicate to Markofski et al., although there were no measurable appetite outcomes (61). The results of the literature search and study selection are reported as a PRISMA flow diagram in Figure 1 (37).

### Characteristics of Included Studies

The characteristics of each of the included reports are summarized in Supplementary Table 3. All identified studies were randomized controlled trials (RCT), apart from one quasi-randomized control trial (54, 61) and one counterbalanced cross-over study (51). A single study implemented an acute exercise bout (51); the remaining 12 interventions ranged in duration from 8 to 44 weeks (46–50, 52–58, 61, 65). Studies utilized a variety of exercise or physical activity interventions with varied frequency and intensity; for the purpose of this review, they were summarized as resistance training [*N* = *5* (47, 49, 51, 58, 65)], aerobic exercise [*N* = *3* (46, 55–57)], or mixed modality [*N* = *5* (48, 50, 52–54, 61)]. In three studies, participants were specifically instructed to maintain their normal diets (47, 48, 65). Six studies excluded obese participants (46–48, 53, 55, 56, 58).

Across the 13 studies, 443 total participants were included in final analyses. Of these, 245 subjects were part of an exercise group and 178 were in a control group. The remaining 20 participants were part of crossover study where they served as the intervention and control; these 20 participants' data were not meta-analyzed to avoid double counting (51). Included subjects had a mean age of 68.9 years (SD = 5.2) and were predominantly female (82.3%). The 13 distinct studies were conducted in 6 countries: 6 in the United States (46, 48, 53–55, 58, 61, 65); 2 in the United Kingdom (50, 51); 2 in Brazil (47, 57); and 1 each in Greece (49), South Korea (52), and Japan (56).

More than one exercise intervention group was described in four reports (49, 50, 57, 58). As previously illustrated in the method, the intervention groups in each of these articles were merged into four respective combined groups; this resulted in a single exercising intervention group and a single control group for each report. Development of the consolidated groups required combination of: (1) low, moderate, and high intensity exercise (49), (2) sedentary behavior fragmentation and light intensity activity (50), (3) dancing and walking interventions (57), and (4) high intensity and low intensity exercise (58).

Only 3 manuscripts provided change scores (48, 51, 53), while no studies provided a correlation coefficient (*r* value) in the absence of a change score. As such, an estimated *r* value of 0.5 was used to calculate change scores for 11 studies (46, 47, 49, 50, 52, 54–58, 65). Because of the small number of available change scores, no sensitivity analyses were performed to assess the effects of either the estimated data or imputed *r* value.

### Risk of Bias of Included Studies

The risk of bias scores for included studies are reported in Supplementary Table 4. The risk of bias was generally “good” for the 15 included reports, with the average score equaling 6.4 (SD = 0.88) out of 11. Risk scores ranged from the upper limit of “fair” equaling 5 at the lowest (48, 54, 61), to the upper limit of “good” equaling 8 at the highest (50). In general, on items related to subject description, outcome reporting, and randomization, all included articles performed adequately. Specifically, for items related to subject eligibility and outcome reporting (PEDro #4, 10-11), all reports scored positively; between 11 and 14 articles earned points for randomization, baseline reporting, and subject measurements (PEDro #2, 4, 8-9). Conversely, reports failed to score on items related to (1) allocation concealment, (2) blinding of subjects, (3) blinding of therapists/administrators, and (4) blinding of assessors (PEDro #3, 5-7).

### Meta-Analysis

Quantitative values for energy intake (*N* = 6), ratings of appetite (*N* = 1), glucose (*N* = 5), leptin (*N* = 5), and adiponectin (*N* = 3) were identified within the studies. Meta analyses with effect size calculations were possible for energy intake (46–48, 50, 55, 58), adiponectin (49, 54, 56, 61), glucose (49, 52, 53, 56, 57), and leptin (49, 52–54, 61, 65). In applicable studies, energy intake was reported in kcal/day (48, 50, 55, 58) and kcal/day/kg (47) of body mass. Glucose was reported as fasting glucose in mg/dl (52, 56, 57) and mmol/L (49, 53). Serum leptin and high molecular weight adiponectin were reported across studies as values of ng/ml and μg/ml, respectively. Because some outcomes were measured in different units across trials, the SMD was calculated for all measures. Ultimately, 18 individual effect sizes were calculated, in addition to the 4 random pooled effects (SMDs) for each outcome of interest. Sub-analyses produced an additional 4 SMDs.

The meta-analysis for energy intake included data from 5 studies and 177 subjects (46–48, 50, 58). Average PEDro score for included studies was “good”, equaling 6.6 (SD = 1.02). Despite homogeneity amongst the studies (*I*^2^= 0%), no significant intervention effect was identified. Sub-analyses on studies which only included non-obese participants were similarly non-significant for any effect of the intervention on energy intake (46–48, 55, 58). Similarly, meta-analysis of adiponectin data revealed no significant intervention effect. Analyses of adiponectin included results from 3 studies with a total of 140 participants and a “good” overall PEDro score (*M* = 6, SD = 0.82); contrary to energy intake, there was very high heterogeneity between these studies (*I*^2^= 92%) (49, 54, 56). Forest plots for energy intake and adiponectin are available in Supplementary Figures 1, 2.

**Figure 2:**
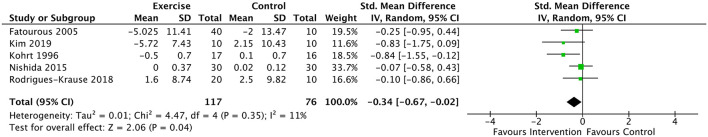
Forest plot for the effect of exercise on glucose. Results favor lower scores in experimental group. Model is statistically significant with low heterogeneity (*I*^2^= 11%), suggesting reliability of the small effect size (−0.34).

Significant pooled effects were noted for both glucose and leptin in favor of the intervention (exercise/physical activity) group. Data from 5 studies and 193 participants were included for glucose estimates (49, 52, 53, 56, 57). Average PEDro score for included studies was ‘good', equaling 6.4 (SD = 0.45). Overall, fasting glucose significantly decreased in the intervention groups compared to the control groups [SMD = −0.34 (95% CI: −0.67, −0.02), *p* < 0.05]. Low heterogeneity (*I*^2^= 11%) was identified across studies (Figure 2). Too few studies including only non-obese participants were identified to sub-analyze the healthy-weight population; sub-analyses with the 3 studies which included obese participants [*N* = 100 participants; (49, 55, 58)] revealed no significant effect of exercise/physical activity on fasting glucose [SMD = −0.33 (95% CI: −0.78, 0.11), *p* = 0.14; *I*^2^= 0%].

**Figure 3:**
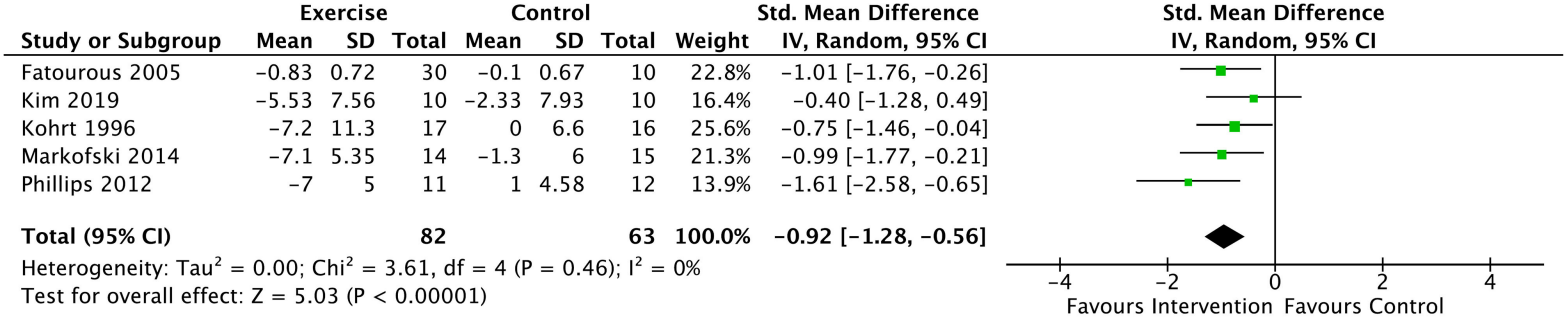
Forest plot for the effect of exercise on leptin. Results favor lower scores in experimental group. Model is statistically significant and homogeneous, suggesting reliability of the large effect size (−0.92).

Analyses of serum leptin included data from 5 studies and 155 subjects (49, 52–54, 61, 65). Average PEDro score for included studies was “good”, equaling 6.2 (SD = 0.75). Results revealed a significant decrease in serum leptin in the intervention group compared to the control, with a large negative effect [SMD = −0.92 (95% CI: −1.28, −0.57), *p* < 0.00001] and no detectible heterogeneity (*I*^2^= 0%) (Figure 3). Subgroup analyses were conducted on these results to remove Markofski, the only study which included a fit, physically active control (54, 61). This subgroup analysis effectively doubled as a sensitivity analysis, removing the only report with a PEDro score <6.0 (5.0). The modified results similarly suggested a large negative effect of exercise/physical activity on serum leptin [SMD = −0.91 (95% CI: −1.31, −0.51), *p* < 0.0001] with low heterogeneity (*I*^2^= 16%). Additional subgroup analyses were conducted to remove Phillips (2012), as all participants were morbidly obese, and some cardiovascular co-morbidity without metabolic disease was indicated (65). Without Phillips (65), the large negative effect on leptin with low heterogeneity was retained both with and without inclusion of Markofski (54): [SMD = −0.81 (95% CI: −1.20, −0.42), *p* < 0.00001], (*I*^2^= 0%) and [SMD = −0.75 (95% CI: −1.20, −0.31), *p* < 0.001], (*I*^2^=0%) respectively. Sub-analyses of the 4 studies which included obese participants (*N* = 112 participants; (49, 52, 54, 61, 65) revealed a significant, large negative effect [SMD = −0.98 (95% CI: −1.39, −0.57), *p* < 0.00001] with low heterogeneity (*I*^2^= 10%).

Visual examination of the random effects funnel plots for glucose and leptin (Supplementary Figures 3, 4) suggested that study distribution was symmetrical for leptin, but some publication bias may exist for glucose. However, no publication bias was identified by the rank correlation test for funnel plot asymmetry for either group. Rosenthal's fail-safe N values estimate the number of non-significant studies required to make the results of analyses non-significant (42); the fail-safe N for glucose was *N* = 6 (*p* < 0.01) while fail safe N for leptin was *N* = 45 (*p* < 0.0001).

Although not specifically identified for meta-analysis, weight (kilogram) change score was extracted from 9 studies (Table 1). No significant effect for this metric was identified. Sensitivity analyses revealed high heterogeneity between studies.

**Table 1:** Weight (kg) change score.

**Article, Year**	**N: Control, Intervention**	**Control**	**Intervention**
Butterworth et al. (46); Nieman et al. (55)	16, 14	2.6 ± 2.4	−0.4 ± 3.5
Fatourous et al. (49)	10, 40	0.0 ± 8.8	1.5 ± 9.6
Kim et al. (52)	10, 10	−0.7 ± 5.0	1.5 ± 4.0
Kohrt et al. (53)	16, 17	0.4 ± 1.7	−2.1 ± 2.2
Markofski et al. (54); Timmerman et al. (61)	15, 15	0.5 ± 9.6	−0.8 ± 19.4
Nishida et al. (56)	31, 31	−0.01 ± 1.1	−1.21 ± 1.3
Phillips et al. (65)	12, 11	−0.1 ± 13.1	−0.1 ± 8.7
Rodrigues-Krause et al. (57)	10, 20	0.8 ± 10.3	0.8 ± 11.1
Taaffe et al. (58)	11, 21	0.2 ± 8.3	0.2 ± 7.1

*Note: Control and Intervention values = M SD; Estimates not possible for (47, 48, 50, 51)*.

No mediation or meta-regression analyses were conducted due to the small number of included studies. This precluded investigation of the varying effects of weight (fat mass) change, age, and exercise intervention on appetite markers.

## Discussion

This systematic review with meta-analysis investigated the effects of exercise and physical activity on appetite regulation in older adults. The findings from this study suggest that exercise/physical activity, summed across modalities/intensities/durations and without concomitant dietary or lifestyle intervention, decreases fasting glucose and serum leptin in adults aged 60+. These changes are reflective of subjects' improved body composition and metabolism and may also correspond with increased satiety sensitivity in trained populations. This suggests that activity alone may provide a meaningful intervention to disease, in part, in this population. However, this study also identified a null effect of exercise and physical activity on adiponectin levels, dietary energy intake, and body weight associated with exercise training. These results are contradictory. Given the results in fasting glucose and serum leptin, increased fasting adiponectin levels were expected, as they are also suggestive of greater satiety sensitivity. However, no such effect on adiponectin was identified. Further, improvements in satiety sensitivity and appetite control should have induced greater changes in energy intake and body weight, but no significant effect on the exercising group over the control group was revealed. The absence of these anticipated changes to energy intake and weight have been similarly noted in previous reviews (13, 35).

Considering the significant effects of exercise on appetite, a large body of evidence supports that healthy levels of glucose and leptin are critical to maximizing fat-mass control, type-2 diabetes prevention, cardiovascular risk reduction, and improved quality of life (36, 66–69). In line with this study, existing research supports that improved exercise fidelity may help to independently reduce fasting glucose and leptin (13, 23). Although short-term reductions in these markers have been associated with compensatory eating by way of increased appetite and decreased activity, maintenance of these reductions has been shown to improve satiety sensitivity and increase energy expenditure (13, 23, 70). Increased satiation and activity may aid in risk reduction and weight maintenance, while the effects of body mass reductions may cyclically improve satiety sensitivity and fasting glucose (13, 23, 71). Of note, the relationships between exercise, physical activity, and appetite outcomes may be affected by the intensity or type of intervention, as well as participants' varying age, levels of fitness, and physiology (e.g., biological responsiveness) (18, 19, 23, 31). However, this review cannot make definitive conclusions about the mediating effects of these and confounding factors (e.g., weight, sex) although the absence of significant weight changes may suggest that the reductions in glucose and leptin are independent of changes in body composition.

The absence of a significant effect on adiponectin identified in this review was unexpected, as increased levels would align with effects on leptin and glucose, suggesting improved satiety (14, 72). Increases in adiponectin have been previously demonstrated in exercise interventions (54, 72, 73), although some evidence suggests there may be no mean effect (74–76). Adiponectin is negatively correlated with fat mass such that overweight/obesity reduces circulating levels (14). The absence of a mean effect may suggest that the insignificant weight changes identified correspond to negligible changes to fat mass and body composition, and that exercise and physical activity alone are insufficient to significantly increase circulating adiponectin levels in the absence of fat-mass reductions (14, 77). However, previous research has supported that exercise alone increases adiponectin in the absence of body composition changes (14, 72), including two of the three studies in this analysis (49, 54). Thus, it may be more likely that this null effect is a result of the small number of studies, and/or is attenuated by additional confounding factors, particularly the varying modalities/intensities/durations of the interventions.

The lack of significant weight change as a result of exercise may also support evidence that exercise and physical activity mitigate the muscle loss associated with reductions in fat-mass by promoting maintenance of fat-free mass (9, 78). Previous research suggests that increased physical activity may improve body composition with no significant effect on body mass by promoting skeletal muscle and reducing adiposity; this may ultimately reduce the risk of sarcopenia and other frailty-related diseases (25, 26, 36, 79). Further, it may be that older adults are more successful at buffering an exercise-induced hunger drive and that exercise or physical activity interventions would provide especially meaningful avenues for fat-mass loss and healthful living in aging populations.

Considering energy intake, identification of no mean effect is supported by recent research and reviews which have noted very little or no post-exercise change in older adults (13, 35, 40). This may be partially attributable to methodology, as some studies specifically instruct participants to maintain their regular diets (47, 48, 65). Additionally, some mechanistic research suggests that the orexigenic effect of exercise is counterbalanced by increased satiety, which may help to reduce compensatory eating in these populations (22). Exercise-induced compensatory energy intake may be further limited within older populations due to the compounding effects of exercise and aging-induced anorexia (8, 12, 13, 80). Reduced gastric emptying and colonic motility, often associated with low activity levels in aging adults, can result in poor nutrition and reduced energy intake (12, 73, 74). However, activity-induced increases in motility may yet promote positive outcomes from improved eating drive and energy intake in some aging adults (81, 82).

### Strengths and Limitations

Although some recent reviews and meta-analyses have probed questions related to exercise and appetite, they have differed significantly from this study in their aim and scope, having included a variety of interventions and measurements, participant ages, and population health statuses (35, 40, 83, 84). As such, to the best of the authors' knowledge, this is the only comprehensive review on the effect of exercise on appetite in healthy older adult populations. A notable strength of this study is that the reports identified in this review were of overall “good” quality (PEDro).

There are several limitations to this study, the primary being that, despite its importance, limited literature is focused on this topic in aging adults. This paucity of available empirical data limits the utility of meta-analyses but highlights the importance of continued investigation. It may be that the relationships between exercise/physical activity and appetite are unique or varied in aging populations, but this has yet to be identified. Further, studies focused on the relationships discussed may still be in progress, unpublished, or unavailable, increasing this review's potential for reporting and publication biases. While the small number of located reports suggests that the true effects of exercise and physical activity interventions on appetite may remain obscured, the identification of studies lacking an intervention effect supports that bias may not be particularly problematic.

Overall, the limited results of this study make confident assessment of bias difficult. Notably, publication bias is difficult to confidently assess in meta-analyses with a small number of included effect sizes (85–88). Methods for assessment are significantly reduced in power and reliability when the number of included studies drops below ten (85–88). Funnel plots are inherently subjective while fail-safe N assumes that all unreported effect sizes are equal to zero (85–88). As such, these results should be interpreted cautiously.

Additionally, because of a lack of available studies, heterogenous reports were compared. For example, all exercise/physical activity interventions were considered equal, despite there being a mixture of modalities, intensities, and durations. Only some studies controlled for diet or utilized managed diets. Similarly, certain studies identified in this review only accrued and reported on one sex, potentially skewing the results. Overall, the small number of studies limited the authors' ability to identify the direct and indirect effects of these confounding variables on the outcomes of interest. The spurious effects of both individual and study characteristics, including weight change, age, sex, and exercise interventions, on appetite markers remain unknown.

Finally, several studies lacked specificity and did not report data meaningful for meta-analysis. Of those which were applicable for quantitative review, many only provided pre-post values as opposed to change scores, and no studies reported correlation coefficients where they would have been appropriate. Change scores and *r* estimations were calculated, which may influence the validity of this study. Limited reporting is similarly reflected by poor performance in some aspects of the risk of bias assessment; although randomized controlled trials were mainly reported on, many favorable study aspects, including allocation concealment and blinding were unclear. Of note, identifying studies including only healthy adults within an older population presents specific challenges, and although this was an aim of the present study, it may be that some manuscripts simply did not report chronic/functional disease status in detail. This is one example of how emphasis on improved reporting methods and data transparency will continue to facilitate accurate aggregation of studies in future meta-analyses.

### Recommendations for Future Research

Future research should aim to increase the body of evidence related to appetite, exercise, and physical activity in aging adults. Although some significant effects were identified within this meta-analysis, a limited number of relatively heterogenous reports were used to draw conclusions. Thus, results should be interpreted cautiously. Additional studies are required to better understand and accurately measure the reported effects. Further investigations should explore the extent, stability, and duration of exercise effects, as well as the feasibility of maintaining these with continued activity. When more data are available, it would be relevant to investigate the independent effects of exercise intensity (vigorous/moderate/light), with consideration of duration, modality, and maintenance of the intervention. Greater investigation of individual characteristics and confounding factors (e.g., age, weight, sex, race, health status, etc.) is also warranted. In future studies, this may be addressed via hierarchical models, mediation analyses (e.g., meta-analyses on indirect effects) or meta-regression. Studies should also seek to explore the differences between appetite regulation in disease-free adults at a normal body weight vs. overweight and underweight populations. It is relevant to further explore the impact of nutrition and diet on the relationships discussed, and to adequately control for any effects.

## Conclusions

Overall, this study suggests the positive effect of exercise and physical activity interventions on some appetite markers but reveals the still-present gap in aging-focused exercise research. Still, the results support the increased prescription of exercise and physical activity for both prevention and treatment of disease across the lifespan. Implementation of an active lifestyle as preventative and reactive medicine may aid in public health measures focused on lengthening disease-free years and reducing the burden of morbidity in later life.

## Data Availability Statement

The original contributions presented in the study are included in the article/Supplementary Material, further inquiries can be directed to the corresponding author/s.

## Author Contributions

JB, KK, and SH contributed to conception and design of the study. SH conducted the article search and selection process, supported by JB. KK assisted in reviewing final article selections. SH performed the statistical analysis and wrote the first draft of the manuscript. JB and KK contributed to sections of the manuscript. All authors contributed to manuscript revision, read, and approved the submitted version.

## Conflict of Interest

The authors declare that the research was conducted in the absence of any commercial or financial relationships that could be construed as a potential conflict of interest.

## Publisher's Note

All claims expressed in this article are solely those of the authors and do not necessarily represent those of their affiliated organizations, or those of the publisher, the editors and the reviewers. Any product that may be evaluated in this article, or claim that may be made by its manufacturer, is not guaranteed or endorsed by the publisher.

## References

[1] United Nations Department of Economic and Social Affairs Population Division. World Population Ageing 2019: Highlights. (2019). Report No.: ST/ESA/SER.A/430. Available online at: https://www.un.org/en/development/desa/population/publications/pdf/ageing/WorldPopulationAgeing2019-Highlights.pdf

[2] Park JH Moon JH Kim HJ Kong MH Oh YH. Sedentary lifestyle: overview of updated evidence of potential health risks. Korean J Fam Med. (2020) 41:365–73. 10.4082/kjfm.20.0165 33242381 PMC7700832

[3] Langhammer B Bergland A Rydwik E. The importance of physical activity exercise among older people. BioMed Res Int. (2018) 2018:1–3. 10.1155/2018/7856823 30627571 PMC6304477

[4] Geneva: World Health Organization. WHO Guidelines on Physical Activity and Sedentary Behaviour [Internet]. 2020 Nov. Report No.: CC BY-NC-SA 3.0 IGO. Available online at: https://www.who.int/publications/i/item/9789240015128

[5] World Health Organization. Physical activity. Health Topics. (2021). Available online at: https://www.who.int/westernpacific/health-topics/physical-activity. (accessed Jun 1, 2021).

[6] Batsis JA Zagaria AB. Addressing obesity in aging patients. Med Clin North Am. (2018) 102:65–85. 10.1016/j.mcna.2017.08.007 29156188 PMC5724972

[7] Malenfant JH Batsis JA. Obesity in the geriatric population – a global health perspective. J Glob Health Rep. (2019) 3:e2019045. 10.29392/joghr.3.e2019045 34027129 PMC8136402

[8] Wysokiński A Sobów T Kłoszewska I Kostka T. Mechanisms of the anorexia of aging—a review. Age. (2015) 37:81. 10.1007/s11357-015-9821-x 26232135 PMC5005824

[9] Gill LE Bartels SJ Batsis JA. Weight management in older adults. Curr Obes Rep. (2015) 4:379–88. 10.1007/s13679-015-0161-z 26627496 PMC5387759

[10] Jacobson A Green E Murphy C. Age-related functional changes in gustatory and reward processing regions: an fMRI study. NeuroImage. (2010) 53:602–10. 10.1016/j.neuroimage.2010.05.012 20472070 PMC3121194

[11] Roberts SB Rosenberg I. Nutrition and aging: changes in the regulation of energy metabolism with aging. Physiol Rev. (2006) 86:651–67. 10.1152/physrev.00019.2005 16601270

[12] Landi F Calvani R Tosato M Martone AM Ortolani E Savera G . Anorexia of aging: risk factors, consequences, and potential treatments. Nutrients. (2016) 8:69. 10.3390/nu8020069 26828516 PMC4772033

[13] Dorling J Broom DR Burns SF Clayton DJ Deighton K James LJ . Acute and chronic effects of exercise on appetite, energy intake, and appetite-related hormones: the modulating effect of adiposity, sex, and habitual physical activity. Nutrients. (2018) 10:1140. 10.3390/nu10091140 30131457 PMC6164815

[14] Becic T Studenik C Hoffmann G. Exercise increases adiponectin and reduces leptin levels in prediabetic and diabetic individuals: systematic review and meta-analysis of randomized controlled trials. Med Sci. (2018) 6:97. 10.3390/medsci6040097 30380802 PMC6318757

[15] Golbidi S Laher I. Exercise induced adipokine changes and the metabolic syndrome. J Diabetes Res. (2014) 2014:e726861. 10.1155/2014/726861 24563869 PMC3915640

[16] Finlayson G Bryant E Blundell JE King NA. Acute compensatory eating following exercise is associated with implicit hedonic wanting for food. Physiol Behav. (2009) 97:62–7. 10.1016/j.physbeh.2009.02.002 19419671

[17] Melanson EL Keadle SK Donnelly JE Braun B King NA. Resistance to exercise-induced weight loss: compensatory behavioral adaptations. Med Sci Sports Exerc. (2013) 45:1600–9. 10.1249/MSS.0b013e31828ba942 23470300 PMC3696411

[18] Blundell JE Gibbons C Caudwell P Finlayson G Hopkins M. Appetite control and energy balance: impact of exercise. Obes Rev Off J Int Assoc Study Obes. (2015) 16:67–76. 10.1111/obr.12257 25614205

[19] Beaulieu K Hopkins M Blundell J Finlayson G. Homeostatic and non-homeostatic appetite control along the spectrum of physical activity levels: an updated perspective. Physiol Behav. (2018) 192:23–9. 10.1016/j.physbeh.2017.12.032 29289613

[20] Davidson TL Jones S Roy M Stevenson RJ. The cognitive control of eating and body weight: it's more than what you think. Front Psychol. (2019) 10:62. 10.3389/fpsyg.2019.00062 30814963 PMC6381074

[21] Van Walleghen EL Orr JS Gentile CL Davy KP Davy BM. Habitual physical activity differentially affects acute and short-term energy intake regulation in young and older adults. Int J Obes. (2007) 31:1277–85. 10.1038/sj.ijo.0803579 17342074

[22] King NA Caudwell PP Hopkins M Stubbs JR Naslund E Blundell JE. Dual-process action of exercise on appetite control: increase in orexigenic drive but improvement in meal-induced satiety. Am J Clin Nutr. (2009) 90:921–7. 10.3945/ajcn.2009.27706 19675105

[23] Beaulieu K Blundell J. The psychobiology of hunger – a scientific perspective. Topoi. (2021) 40:565–74. 10.1007/s11245-020-09724-z

[24] Martins C Morgan L Truby H. A review of the effects of exercise on appetite regulation: an obesity perspective. Int J Obes. (2008) 32:1337–47. 10.1038/ijo.2008.98 18607378

[25] Akehurst E Scott D Rodriguez JP Gonzalez CA Murphy J Mccarthy H . Associations of sarcopenia components with physical activity and nutrition in Australian older adults performing exercise training. BMC Geriatr. (2021) 21:1–10. 10.1186/s12877-021-02212-y 33902464 PMC8077926

[26] Oliveira JS Pinheiro MB Fairhall N Walsh S Franks TC Kwok W . Evidence on physical activity and the prevention of frailty and sarcopenia among older people: a systematic review to inform the world health organization physical activity guidelines. J Phys Act Health. (2020) 17:1247–58. 10.1123/jpah.2020-0323 32781432

[27] Fiatarone MA O'Neill EF Ryan ND Clements KM Solares GR Nelson ME . Exercise training and nutritional supplementation for physical frailty in very elderly people. N Engl J Med. (1994) 330:1769–75. 10.1056/NEJM199406233302501 8190152

[28] de Jong N Paw MJM de Groot LCP Hiddink GJ van Staveren WA. Dietary supplements and physical exercise affecting bone and body composition in frail elderly persons. Am J Public Health. (2000) 90:947–54. 10.2105/AJPH.90.6.947 10846514 PMC1446257

[29] Blundell JE Stubbs RJ Hughes DA Whybrow S King NA. Cross talk between physical activity and appetite control: does physical activity stimulate appetite? Proc Nutr Soc. (2003) 62:651–61. 10.1079/PNS2003286 14692601

[30] Shahar DR Yu B Houston DK Kritchevsky SB Lee JS Rubin SM . Dietary factors in relation to daily activity energy expenditure and mortality among older adults. J Nutr Health Aging. (2009) 13:414–20. 10.1007/s12603-009-0077-y 19390747 PMC2757288

[31] Apolzan JW Flynn MG McFarlin BK Campbell WW. Age and physical activity status effects on appetite and mood state in older humans. Appl Physiol Nutr Metab. (2009) 34:203–11. 10.1139/H08-150 19370051 PMC4306339

[32] Apolzan JW Leidy HJ Mattes RD Campbell WW. Effects of food form on food intake and postprandial appetite sensations, glucose and endocrine responses, and energy expenditure in resistance trained v. sedentary older adults. Br J Nutr. (2011) 106:1107–16. 10.1017/S0007114511001310 21492495 PMC3885865

[33] McGlory C van Vliet S Stokes T Mittendorfer B Phillips SM. The impact of exercise and nutrition on the regulation of skeletal muscle mass. J Physiol. (2019) 597:1251–8. 10.1113/JP275443 30010196 PMC6395419

[34] Drewnowski A Evans WJ. Nutrition, physical activity, and quality of life in older adults: summary. J Gerontol Biol Sci Med Sci. (2001) 56:89–94. 10.1093/gerona/56.suppl_2.89 11730242

[35] Clegg ME Godfrey A. The relationship between physical activity, appetite and energy intake in older adults: A systematic review. Appetite. (2018) 128:145–51. 10.1016/j.appet.2018.05.139 29885385

[36] Pieczyńska A Zasadzka E Trzmiel T Pyda M Pawlaczyk M. The effect of a mixed circuit of aerobic and resistance training on body composition in older adults—retrospective study. Int J Environ Res Public Health. (2021) 18:5608. 10.3390/ijerph18115608 34073970 PMC8197305

[37] Page M McKenzie J Bossuyt P Boutron I Hoffmann T Mulrow C. The PRISMA 2020 statement: an updated guideline for reporting systematic reviews. BMJ. (2021) n71:372. 10.1136/bmj.n71 33782057 PMC8005924

[38] Clarivate Analytics. EndNote. Boston, MA; (2020). Available online at: https://endnote.com/

[39] Corporation for Digital Scholarship. Zotero. Vienna, Virginia; (2021). Available online at: https://www.zotero.org/

[40] Beaulieu K Blundell JE Baak MA van Battista F Busetto L Carraça EV . Effect of exercise training interventions on energy intake and appetite control in adults with overweight or obesity: a systematic review and meta-analysis. Obes Rev. (2021) 5:e13251. 10.1111/obr.13251 33949089 PMC8365695

[41] Higgins J Thomas J Chandler J Cumpston M Li T Page M. editors. Chapter 6: Choosing effect measures computing estimates of effect. In: Cochrane Handbook for Systematic Reviews of Intervention. (2021). Available online at: www.training.cochrane.org/handbook (accessed Jun 5, 2021).

[42] Lipsey M Wilson D. Practical Meta-Analysis. Sage Publications, Applied Social Research Methods Series; vol. 49 (2001).

[43] IBM Corp. IBM SPSS. Statistics for Windows. Armonk, NY. (2020).

[44] The Cochrane Collaboration. Review Manager. 2020.

[45] Hamilton KW Mizumoto A Aydin B. Meta Analysis via Shiny. (2017). Available online at: http://kylehamilton.net/shiny/MAVIS/

[46] Butterworth DE Nieman DC Perkins R Warren BJ Dotson RG. Exercise training and nutrient intake in elderly women. J Am Diet Assoc. (1993) 93:653–7. 10.1016/0002-8223(93)91671-C 8509590

[47] do Nascimento MA Gerage AM Januário RS Pina FL Gobbo LA Mayhew JL . Resistance training with dietary intake maintenance increases strength without altering body composition in older women. J Sports Med Phys Fit. (2018) 58:457–64. 10.23736/S0022-4707.16.06730-X 27735890

[48] Evans EM Van Pelt RE Binder EF Williams DB Ehsani AA Kohrt WM. Effects of HRT and exercise training on insulin action, glucose tolerance, and body composition in older women. J Appl Physiol. (2001) 90:2033–40. 10.1152/jappl.2001.90.6.2033 11356762

[49] Fatouros IG Tournis S Leontsini D Jamurtas AZ Sxina M Thomakos P . Leptin and adiponectin responses in overweight inactive elderly following resistance training and detraining are intensity related. J Clin Endocrinol Metab. (2005) 90:5970–7. 10.1210/jc.2005-0261 16091494

[50] Grant D Tomlinson D Tsintzas K Kolic P Onambele-Pearson G. Displacing sedentary behaviour with light intensity physical activity spontaneously alters habitual macronutrient intake and enhances dietary quality in older females. Nutrients. (2020) 12:2431. 10.3390/nu12082431 32823599 PMC7469014

[51] Johnson KO Mistry N Holliday A Ispoglou T. The effects of an acute resistance exercise bout on appetite and energy intake in healthy older adults. Appetite. (2021) 164:105271. 10.1016/j.appet.2021.105271 33915209

[52] Kim SW Jung WS Park W Park HY. Twelve weeks of combined resistance and aerobic exercise improves cardiometabolic biomarkers and enhances red blood cell hemorheological function in obese older men: a randomized controlled trial. Int J Env Res Public Health. (2019) 16:5020. 10.3390/ijerph16245020 31835508 PMC6950327

[53] Kohrt WM Landt M Birge SJ Jr. Serum leptin levels are reduced in response to exercise training, but not hormone replacement therapy, in older women. J Clin Endocrinol Metab. (1996) 81:3980–5. 10.1210/jc.81.11.3980 8923847

[54] Markofski MM Carrillo AE Timmerman KL Jennings K Coen PM Pence BD . Exercise training modifies ghrelin and adiponectin concentrations and is related to inflammation in older adults. J Gerontol A Biol Sci Med Sci. (2014) 69:675–81. 10.1093/gerona/glt132 24013674 PMC4111637

[55] Nieman DC Warren BJ O'Donnell KA Dotson RG Butterworth DE Henson DA. Physical activity and serum lipids and lipoproteins in elderly women. J Am Geriatr Soc. (1993) 41:1339–44. 10.1111/j.1532-5415.1993.tb06485.x 8227917

[56] Nishida Y Tanaka K Hara M Hirao N Tanaka H Tobina T . Effects of home-based bench step exercise on inflammatory cytokines and lipid profiles in elderly Japanese females: a randomized controlled trial. Arch Gerontol Geriatr. (2015) 61:443–51. 10.1016/j.archger.2015.06.017 26228714

[57] Rodrigues-Krause J Farinha JB Ramis TR Macedo RCO Boeno FP Dos Santos GC . Effects of dancing compared to walking on cardiovascular risk and functional capacity of older women: a randomized controlled trial. Exp Gerontol. (2018) 114:67–77. 10.1016/j.exger.2018.10.015 30389581

[58] Taaffe DR Pruitt L Reim J Butterfield G Marcus R. Effect of sustained resistance training on basal metabolic rate in older women. J Am Geriatr Soc. (1995) 43:465–71. 10.1111/j.1532-5415.1995.tb06090.x 7730525

[59] Fatouros IG Chatzinikolaou A Tournis S Nikolaidis MG Jamurtas AZ Douroudos II . Intensity of resistance exercise determines adipokine and resting energy expenditure responses in overweight elderly individuals. Diabetes Care. (2009) 32:2161–7. 10.2337/dc08-1994 19729520 PMC2782969

[60] Miyashita M Hamada Y Fujihira K Nagayama C Takahashi M Burns SF . Energy replacement diminishes the postprandial triglyceride-lowering effect from accumulated walking in older women. Eur J Nutr. (2020) 59:2261–70. 10.1007/s00394-020-02234-z 32253543

[61] Timmerman KL Flynn MG Coen PM Markofski MM Pence BD. Exercise training-induced lowering of inflammatory (CD14+CD16+) monocytes: a role in the anti-inflammatory influence of exercise? J Leukoc Biol. (2008) 84:1271–8. 10.1189/jlb.0408244 18664531

[62] Coker RH Hays NP Williams RH Brown AD Freeling SA Kortebein PM . Exercise-induced changes in insulin action and glycogen metabolism in elderly adults. Med Sci Sports Exerc. (2006) 38:433–8. 10.1249/01.mss.0000191417.48710.11 16540829

[63] Coker RH Williams RH Kortebein PM Sullivan DH Evans WJ. Influence of exercise intensity on abdominal fat and adiponectin in elderly adults. Metab Syndr Relat Disord. (2009) 7:363–8. 10.1089/met.2008.0060 19196080 PMC3135883

[64] Coker RH Hays NP Kortebein PM Sullivan DH Freeling SA Williams RH . Plasma adiponectin is not altered by moderate or heavy aerobic exercise training in elderly, overweight individuals. Med Sci Sports Exerc. (2006) 38:S189. 10.1249/00005768-200605001-01727 16540829

[65] Phillips MD Patrizi RM Cheek DJ Wooten JS Barbee JJ Mitchell JB. Resistance training reduces subclinical inflammation in obese, postmenopausal women. Med Sci Sports Exerc. (2012) 44:2099–110. 10.1249/MSS.0b013e3182644984 22874536

[66] Schwarz PEH Timpel P Harst L Greaves CJ Ali MK Lambert J . Blood sugar regulation as a key focus for cardiovascular health promotion and prevention: an umbrella review. J Am Coll Cardiol. (2018) 72:1829–44. 10.1016/j.jacc.2018.07.081 30286928 PMC6709577

[67] Zhao S Zhu Y Schultz RD Li N He Z Zhang Z . Partial leptin reduction as an insulin sensitization and weight loss strategy. Cell Metab. (2019) 30:706–19.e6. 10.1016/j.cmet.2019.08.005 31495688 PMC6774814

[68] Fedewa MV Hathaway ED Ward-Ritacco CL Williams TD Dobbs WC. The effect of chronic exercise training on leptin: a systematic review and meta-analysis of randomized controlled trials. Sports Med. (2018) 48:1437–50. 10.1007/s40279-018-0897-1 29582381

[69] Najafipour F Mobasseri M Yavari A Nadrian H Aliasgarzadeh A Mashinchi Abbasi N . Effect of regular exercise training on changes in HbA1c, BMI and VO2max among patients with type 2 diabetes mellitus: an 8-year trial. BMJ Open Diabetes Res Care. (2017) 5:e000414. 10.1136/bmjdrc-2017-000414 29177050 PMC5687538

[70] Kershaw EE Flier JS. Adipose tissue as an endocrine organ. J Clin Endocrinol Metab. (2004) 89:2548–56. 10.1210/jc.2004-0395 15181022

[71] Sirico F Bianco A D'Alicandro G Castaldo C Montagnani S Spera R . Effects of physical exercise on adiponectin, leptin, and inflammatory markers in childhood obesity: systematic review and meta-analysis. Child Obes. (2018) 14:207–17. 10.1089/chi.2017.0269 29762052 PMC5994661

[72] Lin H Hu M Yan Y Zhang H. The effect of exercise on adiponectin and leptin levels in overweight or obese subjects: a meta-analysis of randomized controlled trials. Sport Sci Health. (2017) 13:303–14. 10.1007/s11332-017-0358-5

[73] Yu N Ruan Y Gao X Sun J. Systematic review and meta-analysis of randomized, controlled trials on the effect of exercise on serum leptin and adiponectin in overweight and obese individuals. Horm Metab Res. (2017) 49:164–73. 10.1055/s-0042-121605 28249299

[74] Hayashino Y Jackson JL Hirata T Fukumori N Nakamura F Fukuhara S . Effects of exercise on C-reactive protein, inflammatory cytokine and adipokine in patients with type 2 diabetes: a meta-analysis of randomized controlled trials. Metabolism. (2014) 63:431–40. 10.1016/j.metabol.2013.08.018 24355625

[75] Beavers KM Ambrosius WT Nicklas BJ Rejeski WJ. Independent and combined effects of physical activity and weight loss on inflammatory biomarkers in overweight and obese older adults. J Am Geriatr Soc. (2013) 61:1089–94. 10.1111/jgs.12321 23772804 PMC3714323

[76] Polak J Klimcakova E Moro C Viguerie N Berlan M Hejnova J . Effect of aerobic training on plasma levels and subcutaneous abdominal adipose tissue gene expression of adiponectin, leptin, interleukin 6, and tumor necrosis factor α in obese women. Metabolism. (2006) 55:1375–81. 10.1016/j.metabol.2006.06.008 16979409

[77] Nurnazahiah A Lua PL Shahril MR. Adiponectin, leptin and objectively measured physical activity in adults: a narrative review. Malays J Med Sci MJMS. (2016) 23:7–24. 10.21315/mjms2016.23.6.2 28090175 PMC5181988

[78] Villareal DT Chode S Parimi N Sinacore DR Hilton T Armamento-Villareal R . Weight loss, exercise, or both and physical function in obese older adults. N Engl J Med. (2011) 364:1218–29. 10.1056/NEJMoa1008234 21449785 PMC3114602

[79] Saadeddine D Itani L Kreidieh D El Masri D Tannir H El Ghoch M. Association between levels of physical activity, sarcopenia, type 2 diabetes and the quality of life of elderly people in community dwellings in lebanon. Geriatrics. (2021) 6:28. 10.3390/geriatrics6010028 33803509 PMC8005975

[80] Rosenkilde M Morville T Andersen PR Kjær K Rasmusen H Holst JJ . Inability to match energy intake with energy expenditure at sustained near-maximal rates of energy expenditure in older men during a 14-d cycling expedition. Am J Clin Nutr. (2015) 102:1398–405. 10.3945/ajcn.115.109918 26490491

[81] O'Mahony D O'Leary P Quigley EMM. Aging and intestinal motility: a review of factors that affect intestinal motility in the aged. Drugs Aging. (2002) 19:515–27. 10.2165/00002512-200219070-00005 12182688

[82] Soenen S Rayner CK Horowitz M Jones KL. Gastric emptying in the elderly. Clin Geriatr Med. (2015) 31:339–53. 10.1016/j.cger.2015.04.003 26195094

[83] Johnson KO Shannon OM Matu J Holliday A Ispoglou T Deighton K. Differences in circulating appetite-related hormone concentrations between younger and older adults: a systematic review and meta-analysis. Aging Clin Exp Res. (2020) 32:1233–44. 10.1007/s40520-019-01292-6 31432431 PMC7316693

[84] Giezenaar C Chapman I Luscombe-Marsh N Feinle-Bisset C Horowitz M Soenen S. Ageing is associated with decreases in appetite and energy intake—a meta-analysis in healthy adults. Nutrients. (2016) 8:28. 10.3390/nu8010028 26751475 PMC4728642

[85] Sterne JAC Sutton AJ Ioannidis JPA Terrin N Jones DR Lau J . Recommendations for examining and interpreting funnel plot asymmetry in meta-analyses of randomised controlled trials. BMJ. (2011) 343:d4002. 10.1136/bmj.d4002 21784880

[86] Aert RCM van Wicherts JM Assen MALM van. Publication bias examined in meta-analyses from psychology and medicine: a meta-meta-analysis. PLoS ONE. (2019) 14:e0215052. 10.1371/journal.pone.0215052 30978228 PMC6461282

[87] McShane BB Böckenholt U Hansen KT. Adjusting for publication bias in meta-analysis: an evaluation of selection methods and some cautionary notes. Perspect Psychol Sci. (2016) 11:730–49. 10.1177/1745691616662243 27694467

[88] Higgins J Thomas J Chandler J Cumpston M Li T Page M. editors. Chapter 10: Addressing reporting biases. In: Cochrane Handbook for Systematic Reviews of Intervention. (2021) Available online at: www.training.cochrane.org/handbook. (accessed Jun 5, 2021).

