# Supplementary material for: The Effects of Exercise on Appetite in Older Adults: A Systematic Review and Meta-Analysis
Source: Front Nutr. 2021 Nov 18;8:734267. doi: 10.3389/fnut.2021.734267 (PMC8638160; doi:10.3389/fnut.2021.734267)
Supplement: Supplementary file 1 [file Data_Sheet_1.docx]

Supplementary Material

# Supplementary Table 1. PRISMA 2020 Main Checklist (37)

| **Topic** | **No.** | **Item** | **Location where item is reported** |
| --- | --- | --- | --- |
| **TITLE** |  |  |  |
| **Title** | 1 | Identify the report as a systematic review. | P1 |
| **ABSTRACT** |  |  |  |
| **Abstract** | 2 | See the PRISMA 2020 for Abstracts checklist | Suppl. Table 2 |
| **INTRODUCTION** |  |  |  |
| **Rationale** | 3 | Describe the rationale for the review in the context of existing knowledge. | P2 |
| **Objectives** | 4 | Provide an explicit statement of the objective(s) or question(s) the review addresses. | P2 |
| **METHODS** |  |  |  |
| **Eligibility criteria** | 5 | Specify the inclusion and exclusion criteria for the review and how studies were grouped for the syntheses. | P3 |
| **Information sources** | 6 | Specify all databases, registers, websites, organizations, reference lists and other sources searched or consulted to identify studies. Specify the date when each source was last searched or consulted. | P2-3 |
| **Search strategy** | 7 | Present the full search strategies for all databases, registers and websites, including any filters and limits used. | P3 |
| **Selection process** | 8 | Specify the methods used to decide whether a study met the inclusion criteria of the review, including how many reviewers screened each record and each report retrieved, whether they worked independently, and if applicable, details of automation tools used in the process. | P3 |
| **Data collection process** | 9 | Specify the methods used to collect data from reports, including how many reviewers collected data from each report, whether they worked independently, any processes for obtaining or confirming data from study investigators, and if applicable, details of automation tools used in the process. | P3-4 |
| **Data items** | 10a | List and define all outcomes for which data were sought. Specify whether all results that were compatible with each outcome domain in each study were sought (e.g. for all measures, time points, analyses), and if not, the methods used to decide which results to collect. | P3-4 |
|  | 10b | List and define all other variables for which data were sought (e.g. participant and intervention characteristics, funding sources). Describe any assumptions made about any missing or unclear information. | P3-4 |
| **Study risk of bias assessment** | 11 | Specify the methods used to assess risk of bias in the included studies, including details of the tool(s) used, how many reviewers assessed each study and whether they worked independently, and if applicable, details of automation tools used in the process. | P4 |
| **Effect measures** | 12 | Specify for each outcome the effect measure(s) (e.g. risk ratio, mean difference) used in the synthesis or presentation of results. | P4 |
| **Synthesis methods** | 13a | Describe the processes used to decide which studies were eligible for each synthesis (e.g. tabulating the study intervention characteristics and comparing against the planned groups for each synthesis (item 5)). | P4 |
|  | 13b | Describe any methods required to prepare the data for presentation or synthesis, such as handling of missing summary statistics, or data conversions. | P4 |
|  | 13c | Describe any methods used to tabulate or visually display results of individual studies and syntheses. | P4-5 |
|  | 13d | Describe any methods used to synthesize results and provide a rationale for the choice(s). If meta-analysis was performed, describe the model(s), method(s) to identify the presence and extent of statistical heterogeneity, and software package(s) used. | P3-4 |
|  | 13e | Describe any methods used to explore possible causes of heterogeneity among study results (e.g. subgroup analysis, meta-regression). | P4 |
|  | 13f | Describe any sensitivity analyses conducted to assess robustness of the synthesized results. | P4 |
| **Reporting bias assessment** | 14 | Describe any methods used to assess risk of bias due to missing results in a synthesis (arising from reporting biases). | P4 |
| **Certainty assessment** | 15 | Describe any methods used to assess certainty (or confidence) in the body of evidence for an outcome. | P4 |
| **RESULTS** |  |  |  |
| **Study selection** | 16a | Describe the results of the search and selection process, from the number of records identified in the search to the number of studies included in the review, ideally using a flow diagram. | Figure 1; P4-5 |
|  | 16b | Cite studies that might appear to meet the inclusion criteria, but which were excluded, and explain why they were excluded. | P4 |
| **Study characteristics** | 17 | Cite each included study and present its characteristics. | Suppl. Table 2; P4-5 |
| **Risk of bias in studies** | 18 | Present assessments of risk of bias for each included study. | Suppl. Table 3; P5 |
| **Results of individual studies** | 19 | For all outcomes, present, for each study: (a) summary statistics for each group (where appropriate) and (b) an effect estimate and its precision (e.g. confidence/credible interval), ideally using structured tables or plots. | Figures 2 & 3; Suppl. Table 3 & 4; Suppl. Figures 1-4; P5-7 |
| **Results of syntheses** | 20a | For each synthesis, briefly summarize the characteristics and risk of bias among contributing studies. | P5-7 |
|  | 20b | Present results of all statistical syntheses conducted. If meta-analysis was done, present for each the summary estimate and its precision (e.g. confidence/credible interval) and measures of statistical heterogeneity. If comparing groups, describe the direction of the effect. | Figures 2 & 3; Suppl. Table 3 & 4; Suppl. Figures 1-4; P5-7 |
|  | 20c | Present results of all investigations of possible causes of heterogeneity among study results. | P6-7 |
|  | 20d | Present results of all sensitivity analyses conducted to assess the robustness of the synthesized results. | P6-7 |
| **Reporting biases** | 21 | Present assessments of risk of bias due to missing results (arising from reporting biases) for each synthesis assessed. | P5-7, Suppl. Figures 3-4 |
| **Certainty of evidence** | 22 | Present assessments of certainty (or confidence) in the body of evidence for each outcome assessed. | P6-7, P8 |
| **DISCUSSION** |  |  |  |
| **Discussion** | 23a | Provide a general interpretation of the results in the context of other evidence. | P7-8 |
|  | 23b | Discuss any limitations of the evidence included in the review. | P8 |
|  | 23c | Discuss any limitations of the review processes used. | P8 |
|  | 23d | Discuss implications of the results for practice, policy, and future research. | P8 |
| **OTHER INFORMATION** |  |  |  |
| **Registration and protocol** | 24a | Provide registration information for the review, including register name and registration number, or state that the review was not registered. | Abstract, P2 |
|  | 24b | Indicate where the review protocol can be accessed, or state that a protocol was not prepared. | P2 |
|  | 24c | Describe and explain any amendments to information provided at registration or in the protocol. | P3 |
| **Support** | 25 | Describe sources of financial or non-financial support for the review, and the role of the funders or sponsors in the review. | P9 |
| **Competing interests** | 26 | Declare any competing interests of review authors. | P11 |
| **Availability of data, code and other materials** | 27 | Report which of the following are publicly available and where they can be found: template data collection forms; data extracted from included studies; data used for all analyses; analytic code; any other materials used in the review. | N/A |

#####

# Supplementary Table 2. PRIMSA Abstract Checklist (37)

| **Topic** | **No.** | **Item** | **Reported?** |
| --- | --- | --- | --- |
| **TITLE** |  |  |  |
| **Title** | 1 | Identify the report as a systematic review. | Yes |
| **BACKGROUND** |  |  |  |
| **Objectives** | 2 | Provide an explicit statement of the main objective(s) or question(s) the review addresses. | Yes |
| **METHODS** |  |  |  |
| **Eligibility criteria** | 3 | Specify the inclusion and exclusion criteria for the review. | Yes |
| **Information sources** | 4 | Specify the information sources (e.g. databases, registers) used to identify studies and the date when each was last searched. | Yes |
| **Risk of bias** | 5 | Specify the methods used to assess risk of bias in the included studies. | Yes |
| **Synthesis of results** | 6 | Specify the methods used to present and synthesize results. | Yes |
| **RESULTS** |  |  |  |
| **Included studies** | 7 | Give the total number of included studies and participants and summarize relevant characteristics of studies. | Yes |
| **Synthesis of results** | 8 | Present results for main outcomes, preferably indicating the number of included studies and participants for each. If meta-analysis was done, report the summary estimate and confidence/credible interval. If comparing groups, indicate the direction of the effect (i.e. which group is favoured). | Yes |
| **DISCUSSION** |  |  |  |
| **Limitations of evidence** | 9 | Provide a brief summary of the limitations of the evidence included in the review (e.g. study risk of bias, inconsistency and imprecision). | Yes |
| **Interpretation** | 10 | Provide a general interpretation of the results and important implications. | Yes |
| **OTHER** |  |  |  |
| **Funding** | 11 | Specify the primary source of funding for the review. | None |
| **Registration** | 12 | Provide the register name and registration number. | Yes |

**Supplementary Table 3.** Characteristics of included studies

| **Author, Year** | **Sample N** | **Age** $\boldsymbol{M(SD)}$ | **Study and Sample** | **Intervention (N)** | **Effects** | **Notes** |
| --- | --- | --- | --- | --- | --- | --- |
| Butterworth 1993 & Nieman 1993 (46,55) | *Randomized*  N=32  *Completed*  N=30 | 73.4 (1.1) | *RCT*  Obese: No  Sedentary: Yes  Female: 100.0% | *Aerobic, 12 weeks*  EX (14): Walking  C (16): Calisthenics | Energy Intake | Included cross-sectional comparison of highly trained matched group |
| do Nascimento 2018 (47) | *Randomized*  N=59  *Completed*  N=44 | 66.4 (4.4) | *RCT*  Obese: No  Sedentary: Yes  Female: 100.0% | *Resistance, 12 weeks*  EX (22): Resistance training  C (22): Stretching | Energy Intake | Instructed to maintain diet |
| Evans 2001 (48) | *Randomized*  N=37  *Completed*  N=37 | 67.5 (5.0) | *RCT*  Obese: No  Sedentary: Yes  Female: 100.0% | *Mixed, 44 weeks*  EX (18): 8 weeks flexibility, 36 weeks aerobic & endurance  C (19): No-Treatment Control | Energy Intake | Hormone replacement therapy used in other study arm; Instructed to maintain diet |
| Fatourous 2005 (49) | *Randomized*  N=57  *Completed*  N=50 | 70.4 (3.7) | *RCT*  Obese: Yes  Sedentary: Yes  Female: 0.0% | *Resistance, 24 weeks*  EX1 (14): Low intensity  EX2 (12): Moderate intensity  EX3 (14): High intensity  C (10): No-Treatment Control | Glucose  AdpN | Detraining protocol with measures at baseline, 24 weeks training, and 24 weeks detraining |
| Grant 2020 (50) | *Randomized*  N=35  *Completed*  N=35 | 73.0 (5.0) | *RCT*  Obese: Yes  Sedentary: Yes  Female: 100.0% | *Mixed, 8 weeks*  EX1 (14): Sedentary behavior fragmentation  EX2 (14): Light intensity  C (7): No-Treatment Control | Energy Intake | EX1: participants instructed to not perform sedentary activity for longer than 30 mins, perform 2 mins of upright light intensity activity for every 30 mins |
| Johnson 2021 (51) | *Randomized*  N=20  *Completed*  N=20 | 68.0 (5.0) | *Counterbalanced Cross-Over*  Obese: Yes  Sedentary: No  Female: 100.0% | *Resistance, Acute 5hour*  (20) Baseline, 30 min rest, standard breakfast, 60 min rest, 60 min rest *OR* resistance exercise, 120 min rest, ad libitum pasta meal | Energy Intake  Appetite | Control: intervention group at rest |
| Kim 2019 (52) | *Randomized*  N=24  *Completed*  N=20 | 68.8 (0.9) | *RCT*  Obese: Yes  Sedentary: Yes  Female: 0.0% | *Mixed, 12 weeks*  EX (10): Resistance & aerobic training  C (10): No-Treatment Control | Glucose Leptin | Obesity defined by Asian BMI scale ($\geq25$) |
| Kohrt 1996 (53) | *Randomized*  N=33  *Completed*  N=33 | 65.5 (3.8) | *RCT*  Obese: No  Sedentary: Yes  Female: 100.0% | *Mixed, 44 weeks*  EX (17): 8 weeks flexibility, 36 weeks aerobic & endurance  C (16): No-Treatment Control | Glucose  Leptin | Hormone replacement therapy used in other study arm |
| Markofski 2014 & Timmerman 2008 (54,61) | *Randomized*  N=30  *Completed*  N=29 | 71.2 (5.0) | *Quasi-RCT*  Obese: Yes  Sedentary: Yes  Female: 71.4% | *Mixed, 12 weeks*  EX (14): Endurance & resistance program  C (15): ­­­ Physically active no-treatment control | Leptin AdpN | Intervention group was participants who reported lowest physical activity at baseline |
| Nishida 2015 (56) | *Randomized*  N=69  *Completed*  N=61 | 70.1 (6.2) | *RCT*  Obese: No  Sedentary: UK  Female: 100.0% | *Aerobic, 12 weeks*  EX (31): Bench step exercise  C (30): No-Treatment Control | Glucose  AdpN | Ancillary to published RCT (Ohta ­­­2008) not available in English |
| Phillips 2012 (65) | *Randomized*  N=26  *Completed*  N=23 | 65.6 (2.6) | *RCT*  Obese: Yes  Sedentary: Yes  Female: 100.0% | *Resistance, 12 weeks*  EX (11): Resistance training  C (12): Stretching and Activities | Leptin | All participants obese or morbidly obese (BMI $30-40)$; Some multimorbidity reported, but no metabolic disease; Instructed to maintain diet |
| Rodrigues-Krause 2018 (57) | *Randomized*  N=30  *Completed*  N=26 | 65.0 (5.6) | *RCT*  Obese: Yes  Sedentary: Yes  Female: 100.0% | *Aerobic, 8 weeks*  EX1 (10): Dancing  EX2 (10): Walking  C (10): Stretching | Glucose | Not all participants completed; N=30 intention to treat analyses |
| Taaffe 1995 (58) | *Randomized*  N=36  *Completed*  N=31 | 68.0 (3.0) | *RCT*  Obese: No  Sedentary: Yes  Female: 100.0% | *Resistance, 15 weeks*  EX1 (10): High Intensity  EX2 (10): Low Intensity  C (11): No-Treatment Control | Energy Intake | 17 participants taking exogenous estrogen |

*Note.* $M(SD\boldsymbol{)}$ = mean (standard deviation); EX = exercise group, C = control group, RCT = randomized control trial; Obese = obese participants were included; Sedentary = sedentary participants were included and/or targeted; UK = unknown/unspecified, AdpN = Adiponectin, RDV = recommended daily value

**Supplementary Table 4.** Physiotherapy Evidence Database (PEDro) risk of bias

| **Author (Year)** | **Eligibility** | **Random allocation** | **Concealed allocation** | **Baseline comparability** | **Blinding subjects** | **Blinding therapist** | **Blinding Assessors** | **Adequate follow-up** | **Intention-to-treat analysis** | **Between-group statistical comparisons** | **Point measures and measures of variability** | **Score** |
| --- | --- | --- | --- | --- | --- | --- | --- | --- | --- | --- | --- | --- |
| Butterworth 1993 (46) | 1 | 1 | 0 | 1 | 0 | 0 | 0 | 1 | 1 | 1 | 1 | **7** |
| do Nascimento 2018 (47) | 1 | 1 | 0 | 1 | 0 | 0 | 0 | 0 | 1 | 1 | 1 | **6** |
| Evans 2001 (48) | 1 | 0 | 0 | 0 | 0 | 0 | 0 | 1 | 1 | 1 | 1 | **5** |
| Fatouros 2005 (49) | 1 | 1 | 0 | 1 | 0 | 0 | 0 | 1 | 1 | 1 | 1 | **7** |
| Grant 2020 (50) | 1 | 1 | 0 | 1 | 1 | 0 | 0 | 1 | 1 | 1 | 1 | **8** |
| Johnson 2021 (51) | 1 | 1 | 0 | 1 | 0 | 0 | 0 | 1 | 1 | 1 | 1 | **7** |
| Kim 2019 (52) | 1 | 1 | 0 | 1 | 0 | 0 | 0 | 0 | 1 | 1 | 1 | **6** |
| Kohrt 1996 (53) | 1 | 0 | 0 | 1 | 0 | 0 | 0 | 1 | 1 | 1 | 1 | **6** |
| Markofski 2014 (54) | 1 | 0 | 0 | 0 | 0 | 0 | 0 | 1 | 1 | 1 | 1 | **5** |
| Nieman 1993 (55) | 1 | 1 | 0 | 1 | 0 | 0 | 0 | 1 | 1 | 1 | 1 | **7** |
| Nishida 2015 (56) | 1 | 1 | 0 | 1 | 0 | 0 | 0 | 1 | 0 | 1 | 1 | **6** |
| Phillips 2012 (65) | 1 | 1 | 0 | 1 | 0 | 0 | 0 | 1 | 1 | 1 | 1 | **7** |
| Rodrigues-Krause 2018 (57) | 1 | 1 | 0 | 1 | 0 | 0 | 0 | 1 | 1 | 1 | 1 | **7** |
| Taaffe 1995 (58) | 1 | 1 | 0 | 1 | 0 | 0 | 0 | 1 | 1 | 1 | 1 | **7** |
| Timmerman 2008 (61) | 1 | 0 | 0 | 0 | 0 | 0 | 0 | 1 | 1 | 1 | 1 | **5** |

*Note.* $1=yes, 0=no$; PEDro risk of bias tool (pedro.org.au)

**Supplementary Figure 1.** Forest plot for the effect of exercise on energy intake. Results are statistically insignificant (p=0.93)

**
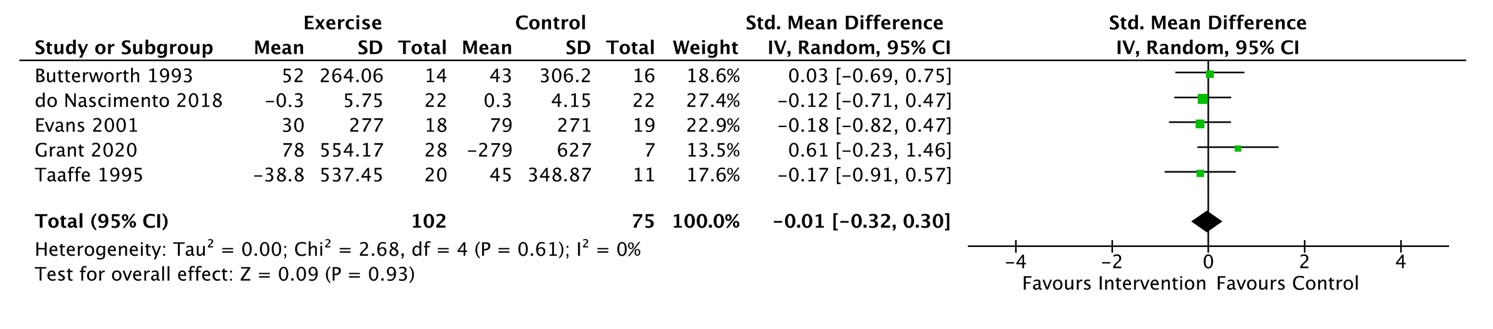
**

**Supplementary Figure 2.** Forest plot for the effect of exercise on adiponectin. Results are statistically insignificant (p=0.15).

**
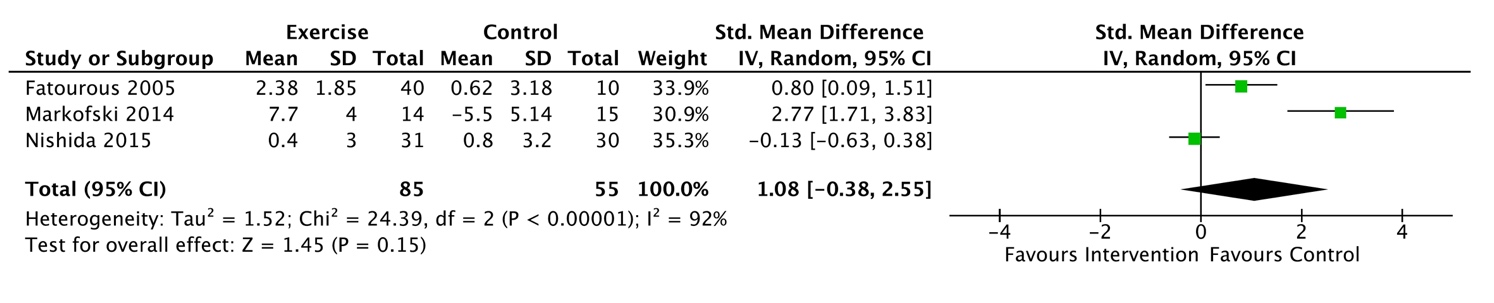
**

**Supplementary Figure 3.** Funnel plot of glucose studies; open circles represent missing null studies estimated with the trim-and-fill method, added into the plot.


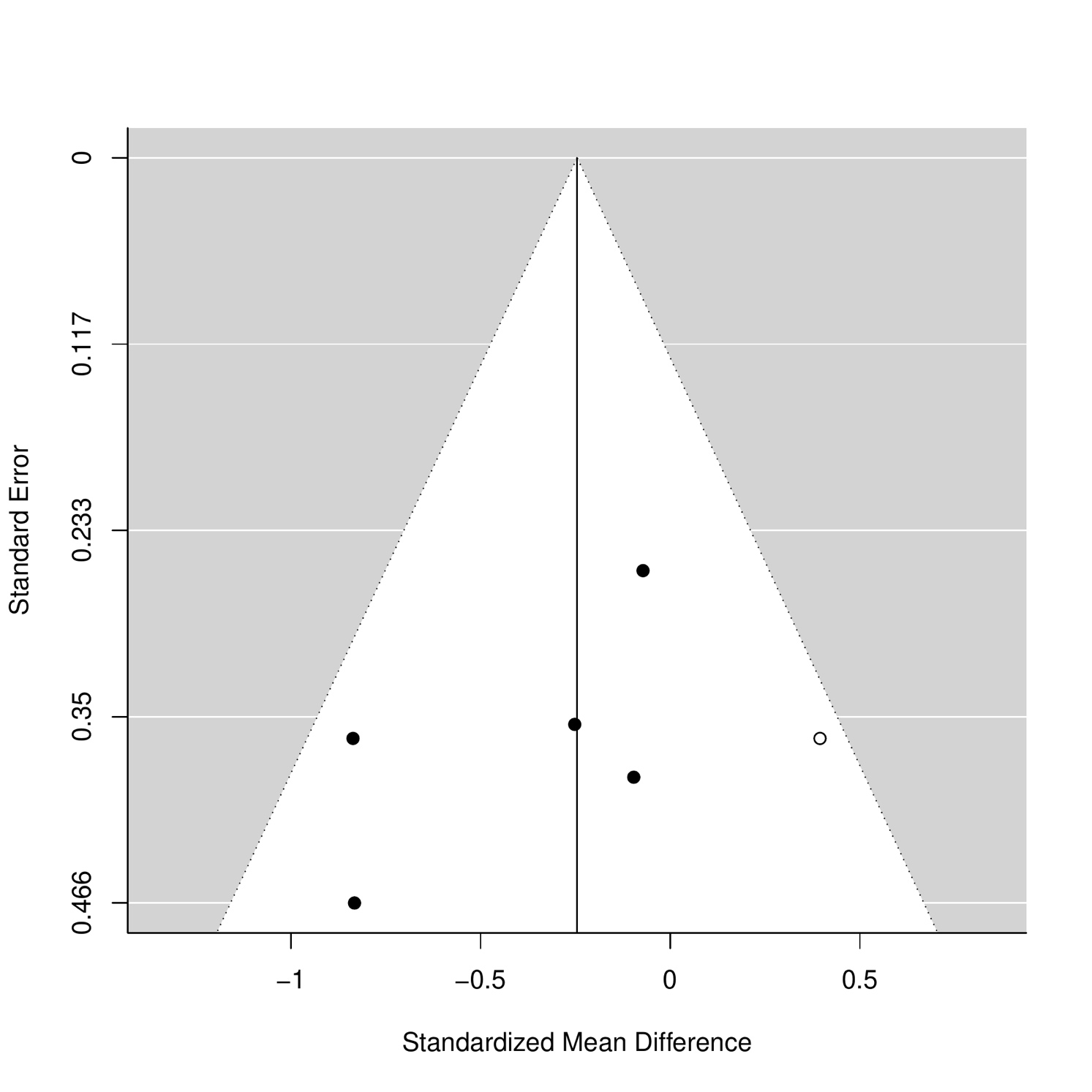


**Supplementary Figure 4.** Funnel plot of leptin studies

**
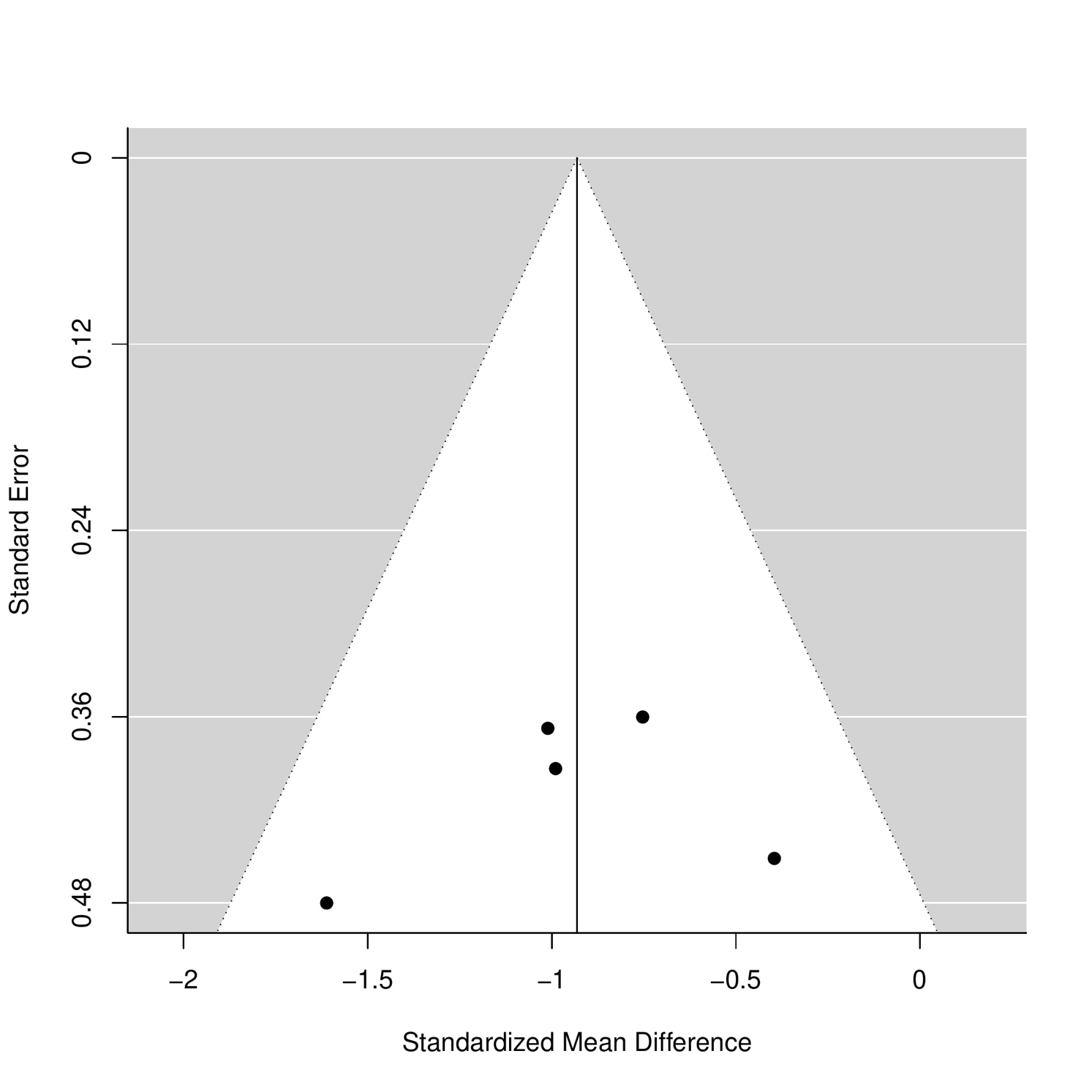
**
